# COVID-19-Induced Left Atrial Appendage Thrombus

**DOI:** 10.7759/cureus.95303

**Published:** 2025-10-24

**Authors:** Steven Douedi, Mihir Odak, Ashraf Sliem, Daniel Ice, Muhammad Raza, Richard Kovach, Chunguang Chen

**Affiliations:** 1 Cardiology, Deborah Heart and Lung Center, Browns Mills, USA; 2 Internal Medicine, Flushing Hospital Medical Center, New York, USA; 3 Cardiovascular Disease, Deborah Heart and Lung Center, Browns Mills, USA

**Keywords:** atrial fib, covid-19, covid-19 hypercoagulability, laa thrombus, watchman device

## Abstract

Left atrial appendage (LAA) thrombus formation is a clinically significant complication that can occur in patients with atrial fibrillation (AF), particularly in the setting of coexisting conditions that affect coagulation. Coronavirus disease 2019 (COVID-19) has been associated with a hypercoagulable state, which may contribute to thrombus formation even in patients on anticoagulation. We describe a patient with persistent AF and recent gastrointestinal and intracranial bleeding who was incidentally found to have COVID-19 infection and was diagnosed with a large LAA thrombus. This case highlights the complex interplay between infection, underlying cardiac arrhythmia, and bleeding risk, emphasizing the importance of early recognition and careful management of thromboembolic risk in high-risk patients.

## Introduction

Coronavirus disease 2019 (COVID-19) has been associated with a wide spectrum of cardiovascular complications, including myocarditis, arrhythmias, acute coronary syndromes, and thromboembolic diseases. One of the most concerning consequences is the development of a hypercoagulable state, which has been linked to both venous and arterial thromboembolic events. The mechanisms are thought to involve systemic inflammation, endothelial injury, platelet activation, and coagulation cascade abnormalities [1].

Venous thromboembolism has been widely described, while arterial complications such as stroke and myocardial infarction have also been reported in patients with COVID-19 [2]. Intracardiac thrombus, however, remains a less well-defined manifestation [3]. The left atrial appendage (LAA) is the most common site of thrombus formation in patients with atrial fibrillation (AF) [4]. Despite anticoagulation, studies have shown that up to 1.6% of AF patients may still harbor LAA thrombi [5]. Recent evidence suggests that patients with persistent AF who develop COVID-19 experience LAA thrombosis more frequently, with rates approximately 2.5 times higher compared to non-infected individuals. Notably, these thrombi are often mural in nature, differing from the typical thrombi usually associated with reduced LAA emptying velocity [3]. Mural thrombi have been observed to respond more favorably to anticoagulation, demonstrating faster resolution than typical thrombi [6].

Given the challenges in balancing anticoagulation with bleeding risk, as well as the potential role of COVID-19 in exacerbating thrombus formation, it is important to recognize this rare but clinically significant entity. We present a case of LAA thrombus identified in a patient with persistent AF and recent gastrointestinal and intracranial hemorrhage who was incidentally found to have COVID-19 infection, underscoring the complex interplay between infection, anticoagulation management, and structural heart interventions.

## Case presentation

An 88-year-old man with a history of coronary artery disease status post multiple drug-eluting stents, aortic stenosis status post surgical aortic valve replacement in 2008 and valve-in-valve transcatheter aortic valve replacement in 2019, hypertension, and permanent AF (CHA₂DS₂-VASc score: 4) presented to an outside facility with black tarry stools. He was compliant with warfarin therapy and maintained therapeutic international normalized ratio (INR) levels (2.30-2.74). Incidentally, he tested positive for COVID-19 by polymerase chain reaction but remained asymptomatic.

During hospitalization, he developed anemia, requiring multiple packed red blood cell transfusions. Gastroenterology evaluated him and performed an esophagogastroduodenoscopy with endoclip placement of a gastric arteriovenous malformation. His hospital course was further complicated by acute mental status changes; a computed tomography scan of the head demonstrated a 3 mm focus of increased attenuation in the right posterior parietal lobe and a small intraparenchymal hemorrhage. Neurology recommended discontinuation of anticoagulation.

**Figure 1:**
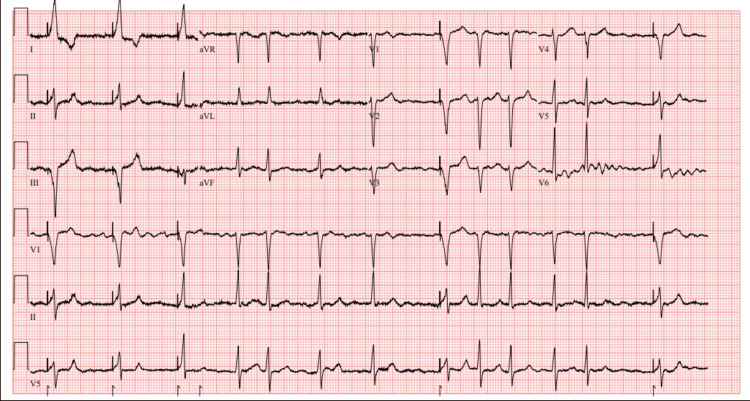
Electrocardiogram showing atrial fibrillation (AF) with intermittent right ventricular paced rhythm

Due to his gastric and intraparenchymal hemorrhage and the recommendation to hold anticoagulation in the setting of permanent AF, the patient was transferred to our facility for Watchman device (Atritech, Plymouth, MN, USA) evaluation. On admission, the electrocardiogram revealed AF with intermittent right ventricular paced rhythm (Figure 1). Laboratory studies, with an INR of 1.13, are shown in Table 1.

**Table 1:** Summary of the patient's lab results

Labs	Value	Reference range
Hemoglobin	10.6 g/dL	12.0-16.0 g/dL
White blood cells	6.2 x 10^3^/mcL	4.8-10.8 x 10^3^/mcL
Platelets	156 x 10^3^/mcL	150-450 x 10^3^/mcL
Blood urea nitrogen	25 mg/dL	6-23 mg/dL
Creatinine	1.1 mg/dL	0.5-1.2 mg/dL
Sodium	139 mmol/L	136-145 mmol/L
Potassium	3.8 mmol/L	3.5-5.1 mmol/L
Alanine aminotransferase (ALT)	20 U/L	0-33 U/L
Aspartate aminotransferase (AST)	32 U/L	5-32 U/L
Prothrombin time (PT)	12.5 seconds	10.0-13.0 seconds
International normalized ratio (INR)	1.13	0.9-1.1

Transthoracic echocardiogram demonstrated a preserved ejection fraction of 55%, severe biatrial enlargement, a well-seated bioprosthetic aortic valve, and moderate mitral regurgitation.

**Figure 2:**
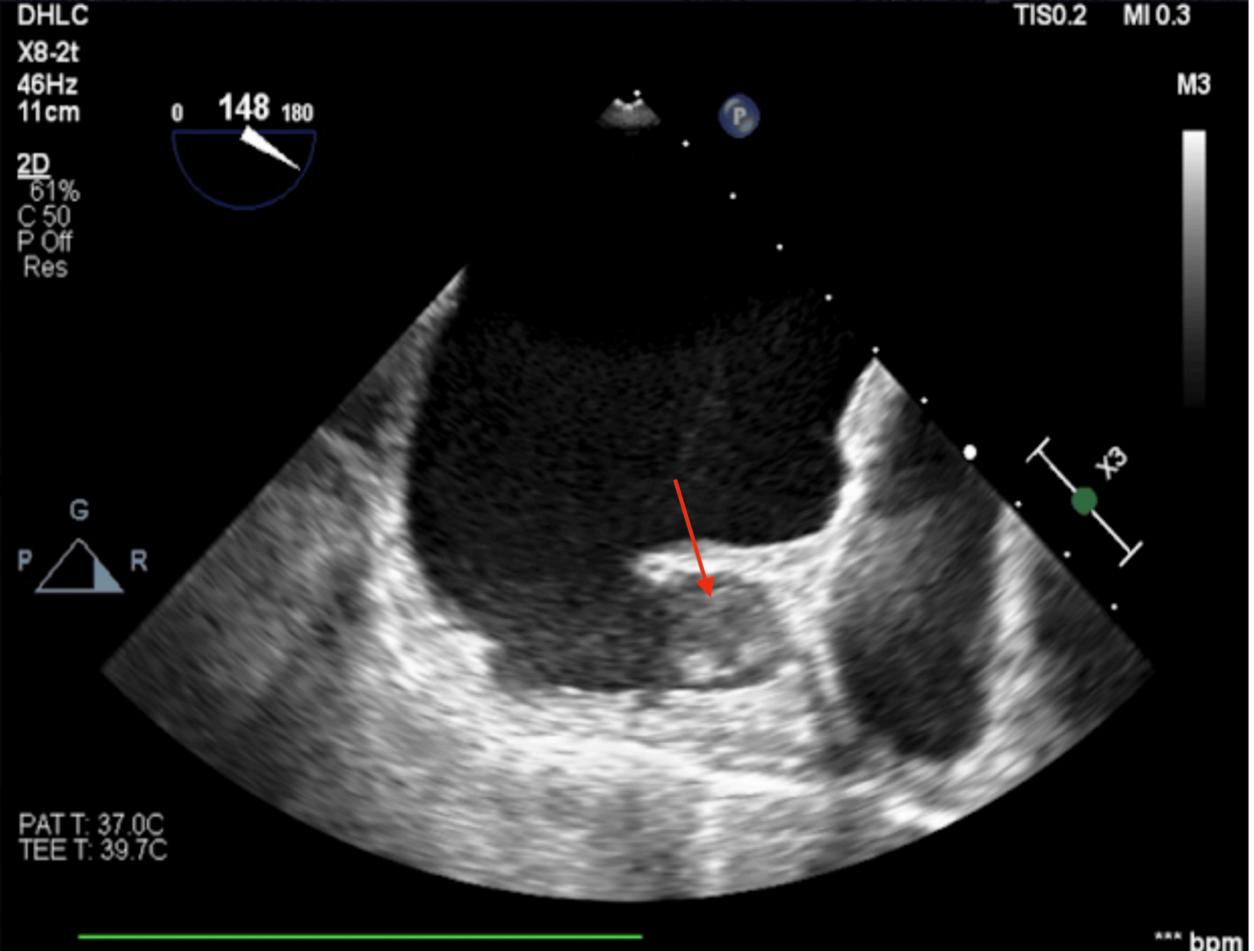
Two-dimensional transesophageal echocardiography (TEE) image of the left atrial appendage showing a 2.5 cm echodensity (red arrow) consistent with thrombus

Transesophageal echocardiogram (TEE) revealed a severely dilated left atrium and appendage (2.9 × 3.1 cm on 2D imaging and 3.2 × 2.0 cm on 3D imaging, with a depth of 2.3-2.4 cm) containing a 2.5 cm echodensity consistent with thrombus (Figure 2).

Due to thrombus identification, Watchman implantation was deferred. Five days later, the patient successfully underwent AngioVac (AngioDynamics, NY, USA) partial thrombus evacuation of the LAA with interatrial septostomy. Attempts for device closure with a 35 mm and subsequently a 31 mm Watchman Flex device were unsuccessful.

The patient remained stable postoperatively and was discharged on day 12 with outpatient follow-up. Anticoagulation was held due to his recent bleeding history, with plans for Amulet device (Abbott, Abbott Park, IL, USA) closure in the near future.

## Discussion

The LAA is a finger-like projection from the body of the left atrium and derives from the primordial left atrium. A “chicken-wing” appearance is the most common LAA shape and accounts for about 48% of morphological types [4]. AF is one of the most common risk factors, with 14% of acute AF patients developing LAA thrombi [7]. This phenomenon is due to stagnation of blood and decreased contraction of the atrium, predisposing to clot formation. While the primary management of LAA thrombi is anticoagulation, studies have found that up to 1.6% of AF patients treated with anticoagulation for one month were found to have LAA thrombi [5]. This emphasizes the importance of imaging, regardless of coagulation status, to identify LAA thrombus.

The primary imaging modality for the diagnosis of LAA thrombus has been strictly defined in the guidelines as TEE [4]. However, with recent advancements, multidetector computed tomography and cardiac magnetic resonance imaging have recently become acceptable alternatives when TEE is unavailable [8]. Once identified with imaging, LAA device closure with an occlusive (such as Watchman device) or exclusive approach under TEE guidance is recommended [4].

While our patient had permanent AF predisposing him to LAA thrombus formation, he additionally had COVID-19 infection, which is known to cause hypercoagulability and the development of arterial thrombosis [3]. Mazur et al. reported larger and more wall-adherent LAA thrombi in patients with AF and COVID-19 than in patients with AF alone [3]. In addition, despite AF and anticoagulation, LAA thrombus has been reported in the literature as an initial finding of COVID-19 infection [6,9]. Despite being adherent to warfarin for anticoagulation with regular INR monitoring, our patient developed a large LAA thrombus. While it is unlikely that COVID-19 is the sole cause of LAA thrombus formation in our case, the viral infection is suggested to play a large role in its formation and appearance.

## Conclusions

COVID-19 infection predisposes patients to a hypercoagulable state, and reports in the literature have identified the virus as a cause of both venous and arterial thrombus formations. While it is unlikely that COVID-19 is the sole cause of LAA thrombus formation, the viral infection is suggested to play a major role in its formation and appearance. COVID-19-induced LAA thrombus is a poorly defined phenomenon that needs further evaluation, as it can potentially have devastating implications on the patient, and, while uncommon, this condition should be promptly recognized and managed. 
